# Knowledge transfer & exchange through social networks: building foundations for a community of practice within tobacco control

**DOI:** 10.1186/1748-5908-1-20

**Published:** 2006-09-25

**Authors:** Cameron D Norman, Tim Huerta

**Affiliations:** 1 Assistant Professor, Department of Public Health Sciences, Faculty of Medicine, University of Toronto, Toronto, ON, Canada; 2 Research Scientist, Provincial Health Services Agency and the British Columbia Child and Family Research Institute, Vancouver, BC Canada; Research Assistant Professor in Health Organizational Management, Rawls College of Business, Texas Tech University, Lubbock, TX, USA

## Abstract

**Background:**

Health services and population health innovations advance when knowledge transfer and exchange (KTE) occurs among researchers, practitioners, policy-makers and consumers using high-quality evidence. However, few KTE models have been evaluated in practice. Communities of practice (CoP) – voluntary, self-organizing, and focused groups of individuals and organizations – may provide one option. This paper outlines an approach to lay the foundation for a CoP within the area of Web-assisted tobacco interventions (WATI). The objectives of the study were to provide a data-driven foundation to inform decisions about organizing a CoP within the geographically diverse, multi-disciplinary WATI group using evaluation and social network methodologies.

**Methods:**

A single-group design was employed using a survey of expectations, knowledge, and interpersonal WATI-related relationships administered prior to a meeting of the WATI group followed by a 3-week post-meeting Web survey to assess short-term impact on learning and networking outcomes.

**Results:**

Twenty-three of 27 WATI attendees (85%) from diverse disciplinary and practice backgrounds completed the baseline survey, with 21 (91%) of those participants completing the three-week follow-up. Participants had modest expectations of the meeting at baseline. A social network map produced from the data illustrated a centralized, yet sparse network comprising of interdisciplinary teams with little trans-sectoral collaboration. Three-week follow-up survey results showed that participants had made new network connections and had actively engaged in KTE activities with WATI members outside their original network.

**Conclusion:**

Data illustrating both the shape and size of the WATI network as well as member's interests and commitment to KTE, when shared and used to frame action steps, can positively influence the motivation to collaborate and create communities of practice. Guiding KTE planning through blending data and theory can create more informed transdisciplinary and trans-sectoral collaboration environments.

## Background

The need to enhance knowledge transfer and exchange (KTE) in health services and population health sciences has been widely articulated [1-3], yet substantial gaps remain in our understanding of the ways innovations transfer into changes in research and practice. The KTE literature meanwhile, reflects a growing body of conceptual models and frameworks with calls for more evaluation research on interventions conducted under real-world conditions [3,4]. Within this corpus, many KTE theories and models have been criticized for not reflecting the multidisciplinary nature of health services and research [5] or considered inadequate for guiding initiatives to translate knowledge into action expeditiously [6,7]. It is therefore important to study KTE in realistic settings, reflecting everyday practice, in order to promote change and foster system improvements. This paper reports on an evaluation of an initiative to foster KTE through an interactive, continuing education model followed by efforts to lay a foundation for development of a formal community of practice. The evaluation is conducted using an innovative blend of behavioural science survey methods with social network analyses as a means of understanding KTE in practice.

The KTE process is guided by an implied hypothesis that suggests: when high quality evidence is placed into a context discernable to others and supports are in place to facilitate the sharing and translation of knowledge developed or gained by some into actionable steps by others – whether it be in research, health care practice, or policy making – that such changes will lead to improvements in the delivery of health care and its outcomes on the population. In order to test such a hypothesis, we must first examine how – and whether – knowledge is shared in the first place between these groups and, if accomplished, answer the question of whether or not that experience has any influence on their behaviour. In doing so, we can begin to understand what impact this might have on consumer health downstream. This paper describes an attempt to partly test this hypothesis in the context of an evaluation of a research meeting intended to promote dialogue, dissemination and network development among a group of researchers, practitioners, and policy makers with interests in Web-assisted tobacco interventions (WATI).

Systematic reviews of KTE and dissemination studies within both practice and research contexts suggest that interventions most likely to influence change use multifaceted approaches simultaneously, provide active educational outreach, or employ interactive delivery methods [4,8,9]. Such findings are congruent with the behavioural science literature that advocates for multi-level, multi-theory interventions aimed at promoting behaviour change at the individual, organizational and systems levels [10-12]. Methodologically, the challenge is to find ways of capturing data about each level and incorporate that into a coherent model of a KTE system of influence within a specific context. The project presented here sought to take up this charge.

In June 2005, a three-day meeting, sponsored by the National Cancer Institute and Health Canada, was held with invited individuals who were known to work in the WATI area by meeting organizers. The purpose of the meeting was to bring together the disparate individuals and organizations working in the area of WATI to share knowledge, explore collaborative opportunities, and develop better practices to guide research, practice and policy activity in this area. One of the intended outcomes of the meeting was develop a network to facilitate KTE beyond the three-day event and explore creating a community of practice (CoP).

### Web-Assisted Tobacco Interventions (WATI)

WATI is a complex and rapidly changing area of tobacco control research and practice. The WATI rubric is applied to the broad application of information technology (e.g., World Wide Web, wireless phone, PDA) to health behaviour change and health promotion interventions designed for smoking prevention and cessation. Some examples include the youth-focussed prevention and cessation website, *The Smoking Zine *[13] and the adult-oriented *QuitNet *program [14].

The application of information technology tools for health promotion, or *behavioural eHealth *(c.f., Norman, 2005 [15]) has been successful at delivering effective behaviour change interventions [16-20]. Given the Internet's reach and availability even small changes attributed to a behavioural eHealth intervention can translate into a large population health effect. Tobacco control is a leading area of behavioural eHealth research [15,21-24] in spite of the challenges in applying standard research models to electronic smoking cessation programs [25,26]. Although WATI use and research has expanded in recent years, there remains a perceived disconnection between members of the community, whereby innovations are developed independently rather than through active sharing of knowledge and collaboration between investigators with complementary expertise. The issue is that of community building and capacity, and something that the organizers of the WATI workshop meeting intended to address.

The WATI community, like tobacco control in general, is composed of a conglomeration of researchers, practitioners, policy makers and consumers/citizens – both individually and in groups – held together by shared interests or foci, rather than an affiliation with a particular discipline or organization. Each individual or group brings a particular knowledge, language, skill base, and set of interests that potentially provide value to the overall enterprise, which only increases in potential impact when these actors are supported in working in a transdisciplinary manner [27]. The task is finding avenues that initiate and sustain collaborative activity among this diverse set of actors.

The WATI meeting was organized by Peter Selby, MD and Scott McIntosh, PhD working with a steering committee of Canadian and US colleagues with interests in eHealth and tobacco control, including funding representatives from the National Cancer Institute in the US and Health Canada. The 2005 meeting served as a follow-up to an initial, smaller meeting held in Toronto in January 2003. Invited participants were not organized or led by any particular body, nor did they share common institutional bonds, roles, or particular disciplinary backgrounds prior to attending the meeting. Although the event was designed to foster collaboration and hopefully seed a network, it was agreed by the steering committee that such a network would have to be self-organized and self-sustaining to succeed in the long term. Given the characteristics of this group, and the aspirations of the organizing committee, it was decided that a community of practice model was an appropriate one to follow. This approach draws on systems thinking, the science of networks and complexity theory [28-31] which explores the behaviour of self-organizing structures. These self-organizing, adaptive, and responsive learning systems use simple rules and procedures to guide collective, transorganizational learning.

Communities of practice are self-organized, voluntary, focused collectives of people and organizations who work toward common understanding on a given issue [32,33]. Communities of practice use resources efficiently, help drive strategy, elucidate and transfer best practices, cultivate partnerships, develop professional skills, and promote rapid dissemination of knowledge within teams and groups with a common purpose [33]. The CoP approach is consistent with systems thinking in that it encourages self-organization and is suited a transorganizational structure that lacks a centralized command. The CoP approach to KTE has garnered attention within tobacco control (e.g., The 2nd Annual Invitational Symposium for Research to Inform Tobacco Control, Canadian Tobacco Control Research Initiative [34]) and is ideally suited to a knowledge environment that is both complex and rapidly changing such as WATI [12,35,36].

By taking a systems approach to KTE, it was also suggested that methods that could tap into systems-level issues within WATI were needed to effectively evaluate the meeting and provide the necessary data that could aid in efforts to create a CoP. The authors (CN & TH) were brought into the advisory group to assist in planning the meeting and conducting the evaluation with this in mind. Bringing backgrounds in public health, community psychology and evaluation (CN) and organizational behaviour and network research (TH), the authors developed an evaluation framework designed to capture the necessary information to support development of a CoP, while also allowing exploration of combined methods of studying a KTE context that could potentially be used in other settings. The caveat was that there were limited resources to conduct the evaluation and so measurement tools needed to be simple and concise.

The evaluation had three aims: 1) to assess the impact of a mixed-method, interactive approach to education and KTE on collaborative activity, 2) to provide an empirical foundation to guide the development of a CoP within this group, and 3) to pilot the implementation of a novel, systems-oriented approach to evaluating KTE using combined evaluation and social networking methodologies.

## Methods

Solomon argues that the future of behaviour change research is translational, interdisciplinary, methodologically innovative and collaborative in nature [37]. Perhaps not surprisingly, similar language has been used to discuss the needs and possible futures for KTE and dissemination research [38]. Research into knowledge practices in firms known for innovation found that KTE takes place within the context of relationships [39], suggesting that it cannot be understood apart from these relational interactions. Best and colleagues go further to argue that these relationships exist within the context of a larger system and suggest that an ecological approach is required to understanding KTE in practice contexts [1]. Given the need to consider the impact of an intervention on both individuals and a system, a new approach was required to understanding KTE.

Variables of interest included: knowledge, attitudes, expectations and learning were assessed using a short instrument developed for this study. Likert scale items measured agreement (e.g., *strongly agree *to *strongly disagree*) on a short set of questions. Factor analyses were conducted on the items in the follow-up survey to create scales related to outcomes (knowledge, expectations, actions, networking, and information seeking) with coefficient alphas considered 'good' using psychometric guidelines [40]. However, analyses presented here were conducted at the item, not scale, level given questions about the reliability of such groupings with the current sample size.

To capture the social aspect of learning and gain an understanding of the bounds of the system, a methodology based on social network analysis [41] was used to develop a relationship map of the group. Participants were asked to identify individuals by name that were a source of information or knowledge about WATI, the nature of that relationship (e.g., contracted or not), and the level of impact that individual had on the subject matter from low to high. When coupled with demographic information collected as part of the survey, the data presents a rich visual tool that can be used to inform decisions.

### Participants

Surveys were distributed to all meeting attendees (N = 27) at the start of the meeting and three weeks after the meeting. Twenty-three participants completed the baseline survey (85% response rate). Of these participants, 13 had attended the first WATI meeting in Toronto. Twenty-one participants (77% of eligible attendees) completed the 20-item 3-week follow-up survey online. A profile of participants is located in Table 1.

**Table 1:** Profile of Baseline Survey Participants

Category	Response	N
Location	United States	13
	Canada	9
	Australasia	2
Discipline	Medicine	3
	Psychology/Psychiatry/Mental Health	10
	Nursing	1
	Public Health Sciences	6
	Biology	1
	Education	1
	Other	1
Work Setting	Hospital	2
	Health Care	2
	Government	6
	Non-Profit/NGO	3
	For-Profit	1
	University	9

### Materials

Baseline data was collected via pencil and paper survey while the three-week follow-up was delivered via the Internet using Surveymonkey[42], a publicly accessible, secure survey platform, prompted by a Web linked delivered to the secure email address provided at baseline.

#### Baseline survey

Participants completed a mixed format survey combining eight multiple choice items on meeting expectations, demographics and WATI research activities and 16 items on five-point Likert scale on importance or confidence ranging from *very *to *not at all*. In the second part of the survey, participants identified up to 10 individuals who were perceived to have influence on their WATI related work including: research collaborators, those whom they share WATI information with, appropriate funding representatives, co-authors, and policy makers. These individuals did not have to be present at the meeting. The procedure was repeated focussing on organizations of influence. This approach has been used with related research networks [43,44] including in tobacco control [45]. For each individual or organization identified, participants were asked to 'type' the intensity of their relationship as either *shared information, team (no contract) *or *team with contract*. Examples of contracts included a funded grant, a formalized project, a panel or committee. Relationships were classified in an ordered hierarchy with the least intense contact form involving *shared information *(i.e., direct active exchange including personal emails as opposed to passive exchange such as listservs and mailing lists). Network data was analysed using UCINet [46] and supported by NetDraw [47].

#### Three-week follow-up

A follow-up survey was sent out three-weeks post-meeting. The survey evaluated perceived impact of the meeting on WATI-related knowledge, KTE activities, and intentions to engage in CoP-building activities. The first 10 questions used a 5-point Likert scale (*strongly agree *to *strongly disagree*) and asked about perceived learning outcomes and intentions to act, while the final 10 questions used a yes/no format to examine follow-up activities. A modified Dillman procedure [48] was used to solicit responses after non-response to the first email survey request. This procedure involves structured messages that are increasingly tailored to the participant sent over time in order to encourage a response.

## Results

### Baseline behaviour data

Simple descriptive statistics were calculated using SPSS 11.5 [49] to determine the relative amount of agreement on each item. Respondents believed it was important that the meeting produce increased collaborations (*mean *= 1.96, *SD *= 0.82), research opportunities (*mean *= 1.91, *SD *= 0.67), and strengthen or initiate relationships with others in WATI (*mean *= 1.43, *SD *= 0.66). Most participants were confident that attending the meeting would expand their network of colleagues (*mean *= 1.65, *SD *= 0.65), although there was doubt whether attendance at the meeting would lead to changes in behaviour (*mean *= 2.04, *SD *= 1.07). There was less optimism that the meeting would influence capacity to conduct WATI research (*mean *= 2.65, *SD *= 1.07). Results are presented in Table 2.

**Table 2:** WATI II Meeting Expectations

Question	Mean (Std Dev)
*1. How important is it that the WATI II meeting produce increased collaboration opportunities for you?*	1.96 (0.82)
*2. How important is it that WATI II meeting produce increased knowledge of research opportunities?*	1.91 (0.67)
*3. How important is it that WATI II strengthen or initiate relationships with others engaged in WATI?*	1.43 (0.66)
*4. How important is it that WATI II leads to the production of a specific product (e.g., manuscript, grant application)?*	2.83 (1.19)
*5. How important is it that WATI II produces collaborations with those outside of my current field of research/practice?*	2.50 (0.86)
*6. How confident are you that the WATI II meeting will expand your collaborative network?*	1.65 (0.65)
*7. How confident are you that the WATI II meeting will produce new knowledge for you in?*	1.30 (0.47)
*8. How confident are you that WATI II will produce changes in your practice/research in the next 6-months?*	2.04 (1.07)
*9. How confident are you that participation in WATI II will increase your capacity for conducting research in the next 6-months?*	2.65 (1.07)
*10. How confident are you that participation in WATI II will increase your capacity to deliver WATI-related interventions?*	2.26 (1.14)
*11. How confident are you that the WATI II meeting will expand your collaborative network?*	1.74 (0.86)
*12. How confident are you that the knowledge produced from the WATI II meeting will be translated or disseminated beyond the WATI community at a later date?*	2.22 (0.80)
*13. How confident are you that the knowledge produced from the WATI II meeting will be translated or disseminated within the WATI community at a later date?*	1.86 (0.99)
*14. How confident are you that the action items that emerge from the WATI II meeting will be acted upon?*	2.27 (0.83)
*15. How confident are you that the WATI II meeting will produce actions that lead to policy changes (e.g., increases in grant opportunities)?*	2.73 (0.88)

Scale:

1 – Very Important/Confident

2 – Somewhat Important/Confident

3 – Neutral

4 – Unimportant/Not Confident

5 – Not at all important/Confident

### Three week follow up

Participants reported increases in overall knowledge of WATI-related research (*mean *= 1.47, SD = 0.51) and resources (*mean *= 1.89, SD = 0.45) and that the meeting met participants' expectations for learning (*mean *= 2.16, *SD *= 0.40) and networking (mean = 1.68, SD = 0.58). Most participants had attempted to contact another meeting attendee or reported having been contacted by someone they met at the event, while 57% of participants who took action of some sort on ideas generated from the meeting (*mean *= 2.36, *SD *= 0.83) demonstrating an impact on KTE beyond the meeting. Follow-up results are summarized in Table 3.

**Table 3:** Three-week Follow-up Outcomes of the WATI II meeting

Question	Mean (SD)
*1. My knowledge of WATI-related research increased*	1.47 (0.51)
*2. My knowledge of WATI-related resources (websites, tools, etc) increased*	1.89 (0.45)
*3. My knowledge of WATI-related better practices increased*	2.52 (0.61)
*4. My knowledge of WATI-related publication or dissemination opportunities increased*	2.26 (0.73)
*5. My learning expectations were met*	2.16 (0.40)
*6. My networking expectations were met*	1.68 (0.58)
*7. My WATI-related work is likely to change as a result of what I learned at WATI II*	2.16 (0.75)
*8. I have taken action on developing ideas that were generated as a result of my experience at the WATI II meeting*	2.36 (0.83)
*9. I met at least one new person that I intend to collaborate with sometime in the next 6 months*	2.15 (1.01)
*10. The meeting was successful in developing a better practices framework for WATI*	2.66 (1.03)

Response options (value):

Strongly Agree = 1

Agree = 2

Neither Agree or Disagree = 3

Disagree = 4

Strongly Disagree = 5

### Network mapping

**Figure 1:**
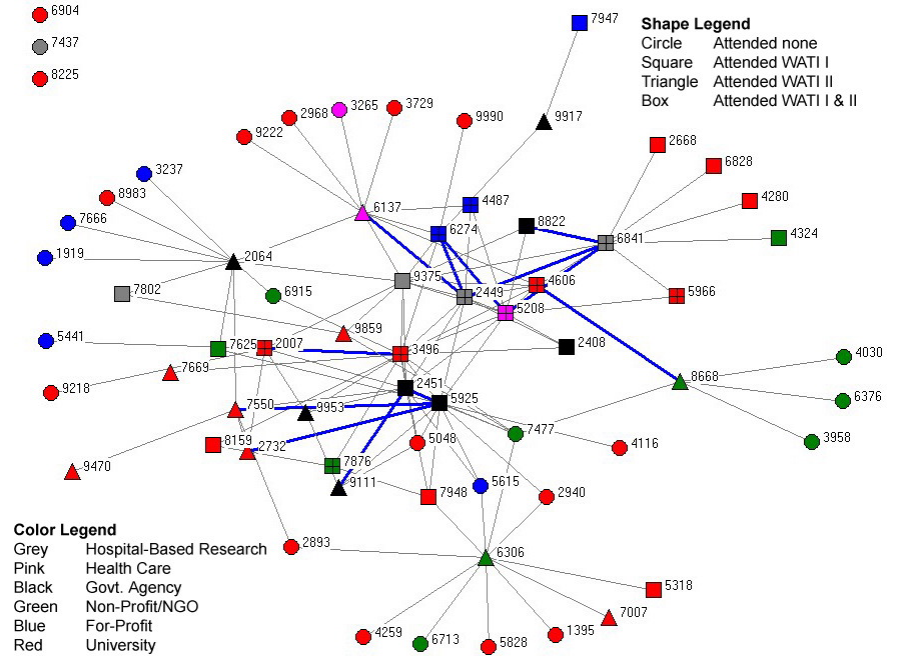
Relationship network map of WATI Community Members.

Network data was compiled using UCINet and NetDraw to produce network maps, which were presented to participants on the second day of the meeting. Figure 1 illustrates the influential relationships among attendees and others in the WATI community using participant data (names removed) presented in the form of four digit numbers. The map has several structural features that require explanation. The colour of the lines provides information on the direction of relationships within the network. Gray lines are unidirectional relationships where a member indicated a relationship that was unconfirmed by the other, which could be due to incomplete data (individual was not present at the meeting). The map illustrates that those most likely to serve a pendant role dominate the edge of the network map. Pendants are those individuals linked to a single person within the network. Blue lines represent confirmed relationships, meaning relationships where both participants have identified each other as a source of information and influence in WATI related endeavours. For example, the map shows a high concentration of people who are linked together at the centre. Shapes are used to indicate whether a participant attended either WATI meeting or was invited or not, while colours indicate the types of institutions that participants were based out of.

Centrality (*Freeman centrality *= 4.188%) is the degree to which relationships are centralized within the network, and can be inferred by position within the network map for each member. The Figure 1 map concentrates those with the greatest number of relationship connections to other connected people towards the centre of the network map. To that end, participant numbers 9375, 2449, 5208 and 3498 were most central to the community. However, those identified by numbers 5048, 5615, 2940, 2893 and 6915 have never been to a WATI meeting, yet are also central to the overall knowledge base. Their absence could have been due to an inability to attend or having not been identified for invitation prior to the meeting.

Both frequency and intensity data on relations was collected, which allow for greater discrimination. Conventional analysis holds that when two individuals indicate a connection between one another, the most common approach is to average these two factors as the strength of the relationship. However, this becomes problematic when one person indicates a weak relationship and the other indicates a strong relationship. For example, using a coding framework with none (0), weak (1), moderate (2) and strong (3), the implication from the data is that a moderate relationship exists [(1+3)/2]. Likewise, if both indicate a moderate relationship, the result would be a value of 2 for the interaction [(2+2)/2]. Using a square root sum of the squares approach discriminates between these two cases [Sqrt[(1*1)+(3*3)] = 3.16] v. [Sqrt[(2*2)+(2*2)] = 2.83] and allows for finer discrimination among differential relationships.

**Figure 2:**
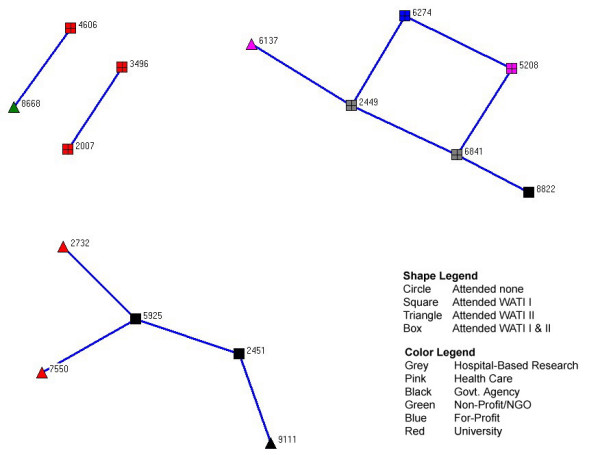
Reciprocal networks among WATI Community Members.

Figure 2 focuses on reciprocal networks – those with the blue connections. This provides the clearest picture of the absence of a true network within the WATI group in terms of research to practice links. Relationships scores were calculated as the product of the square of the strength of the relationship, where shared relationship, team no contract, and team contract were valued as 1, 2, and 3, respectively and the strength of the influence, scored as 1, 2, and 3 for low, medium and high. The resulting individual relationship score varied from 0, indicating no relationship, and 18, indicating a contracted relationship with high impact on WATI related activities. The resulting map illustrates a paucity of translational links connecting teams working in research and practice. This methodology has been used to examine other similar practice networks [45].

**Figure 3:**
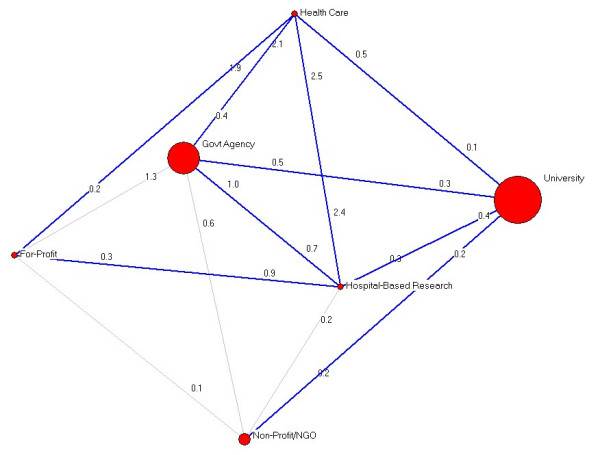
Trans-sectoral networks among the WATI Community.

Figure 3 presents the trans-sectoral network. Individuals were organized according to sector: university, hospital-based research, non-profit/non-governmental organization, governmental agency, for-profit, or health care agencies based on their reported institutional affiliation. Scores were averaged both within and between individuals in these categories. Node sizes in Figure 3 correspond with the number of people within each agency type. Numbers close to each node indicate the strength of the connection (or relationship) between sub-communities. For example, the for-profit community has KTE relationships with health care (0.2) and hospital-based research (0.3).

Figure 3 shows no links between the for- profit and university communities and between health care and the non-profit communities. The network model suggests WATI participants representing governmental organizations occupy a more influential position in the network than most others. Substantively, it should be noted that the network is very diverse in that we see very little clustering of institution types, indicating a strong research-practice relationship within the network. In addition, it is notable that 63% (17 of 27) of the pendants are based in university settings.

### Applying the data to community of practice (CoP) building

Baseline descriptive data indicated that participants had modest expectations for learning, perceived few barriers to action, had both positive expectations and interest in seeing something emerge from the meeting. Network data collected on the first day of the meeting was presented in visual form to the WATI audience on the second day by the authors for use in seeding a discussion on ways in which the group could work together, including building a community of practice. It was the authors' view that the impact of presenting the visualization of the network was highly effective at engaging participants in CoP discussions.

Presentation of the network models provoked a group conversation around three areas. The first was the notable and continued absence of a number of central individuals from the map at the meeting. The need to outreach and include those not in attendance was considered an important step towards building a genuine, inclusive CoP. Unfolding the network to show reciprocal ties was also of interest, but providing an indication of the teams involved in this endeavor, as well as demonstrating the lack of cohesion among most of the network.

When presenting the network models, the authors highlighted the fact that the network map was, in fact, an overlay of ego networks that created an illusion of disconnectedness. Since those on the periphery of the network were not included in the study, participants were reminded that the number of pendants could be misleading as it was impossible to tell how connected they were to the rest of the group, but that if researchers were to enquire with these referees, a more complex network may emerge. Finally, the number of peripheral players who never had attended either of the two WATI meetings struck participants as problematic and a possible cause for intervention in creating the CoP. There were no negative reactions to the presentation of the data. Rather it created an awareness of just how much of the network was not a part of the meeting. It also required the community to come to terms with conflicting definitions of membership as discussion resulted in awareness of the absence of key stakeholders in the meetings.

The discussion yielded approval and interest in exploring a CoP and a nominal group approach was used to develop priority action steps. Among the themes derived from the nominal group was an expressed interest in building a more robust network (i.e., create stronger and a greater number of links between more members of the group). To support this, the group elected to focus on specific activities that could engage group members over the coming months including developing: 1) a recommended minimum data set of items for use in WATI research, 2) a mission statement, 3) a common language for WATI-related technologies and functions, 4) WATI intervention development guidelines, and 5) a strategy to engage consumers. Individuals indicated both their interest in the activity and their willingness to take action on the topic on a sign-up sheet.

The work projects were used to both test the capacity and willingness of WATI attendees to work collaboratively and to build some 'quick wins' that would provide a basis for a more focussed CoP building effort. The authors offered to facilitate the start of this process using email and a project blog [50], but otherwise encouraged work groups to self-organize. The agreed goal was to lay the foundation for developing a CoP that could be built upon the success of these small, informal working groups.

## Discussion

The evaluation of the WATI meeting suggest that there is both the interest and motivation to create a collaborative KTE network within WATI in the form of a CoP. Wenger and colleagues suggest that CoP's develop in five phases [51]. The first phase, *Potential*, involves discovering ideas and people and finding a common ground between interested parties. Phase two, *Coalescing*, is the incubation stage where community members start to act collaboratively and deliver knowledge products. The third phase, *Maturing*, is where the CoP starts achieving focus and a vision for itself, rules and norms get established and a learning culture starts to become evident. *Stewardship *is the fourth phase, where issues of maintaining focus and sustaining momentum and innovation are most salient. The development cycle ends with *Transformation *where the CoP either renews itself by bringing in new ideas or people, changes directions, or dissolves as the needs of the group change.

The WATI meeting signified the *Potential *phase. The baseline data on expectations, the initial network map that illustrated to participants what the WATI network looked like and the ensuing discussion about what it could look like if the group was interested in working collaboratively towards a community practice exemplified this. The three-week follow-up data demonstrated the ability of the WATI group to work together, with many participants reporting having taken some actions as a result of attending the meeting. Through discussions via email with the nascent community over the 6-months that followed, it was evident that group had moved into the *Coalescing *stage demonstrated by the progress made on most of the five tasks and by the establishment of working relationships within and beyond the group to include groups with similar interests (e.g., the North American Quitline Consortium). Following this success, the group seems poised to move forward into the *Maturing *stage.

Baseline data suggested that participants were already accustomed to working across disciplinary boundaries, but not as members of teams where there was evidence of little cross-team interactions, nor were there examples of trans-sectoral collaboration among members of the WATI group. Given that WATI research and practice exists at the intersection between two interdisciplinary fields like tobacco control and eHealth it is not surprising that there was a high-level of interdisciplinary collaboration among WATI members. Low levels of trans-sectoral collaboration might suggest that WATI members are finding common interests within their own or similar physical environments and not venturing further. However, if the WATI group aspires to work more collaboratively across sectors, some form of intervention is needed to create these links, particularly given the group's global dispersion.

A potential solution for this group is implementing the CoP virtually. Much has been written on the establishment and functions of virtual communities [52-56] although, like CoP's, there remains little systematic evaluation evidence on their effectiveness in fostering KTE-related changes. In general, the CoP literature reveals few summative evaluations or examples where a community's development has been guided by empirical data. This study is unique in both its data-driven approach to CoP development and in the methodological approach used to gather the data itself by blending both traditional analytic tools and theories (e.g., self-efficacy, behavioural intentions) with social network analytic methods.

## Limitations

Presentation of the behavioural and social network data to the WATI II attendees for feedback provided a form of validation for the baseline measures; however it does not replace the need for a formal psychometric assessment of our instruments. Although factor analyses conducted on the 3-week follow-up data suggest that the scales developed for the study were sound, the small sample size limits the conclusions that can be drawn. The sample size also limited the analyses that could be reasonably performed overall and the explanatory power regarding claims about the entire network given that the network map identified people outside of the WATI meeting as significant sources of influence. Social network analyses are optimized when a total population sample is achieved, particularly in cases where networks are small and specialized such as this one and accompanied by an assertive follow-up strategy [45]. The requirement for exhaustive network coverage makes this methodology prohibitive for use in large networks unless non-specific data is acceptable.

There are also limitations to the community of practice approach itself. Self-organized networks, like a CoP, require commitment and investment of resources from a large number of committed, engaged members of the community who often receive little compensation for their involvement. Without broad investment across the network, there is increased susceptibility to it falling apart should key individuals leave the network. Collective action requires leadership and resources, and without a centralized command or individual responsible for the network, they can easily dissolve, particularly if they fail to provide the knowledge value that community members expect. In this study, we discovered that many potential members of this community were identified as not present at the meeting. If a representative community is to be established, these individuals on the periphery of the network need to be engaged in the CoP initiative.

For a self-organizing, adaptive, and responsive learning system such as a CoP to succeed, it must engender broad engagement and enlist leadership. At the end of the WATI meeting, attendees identified simple, actionable activities that could strengthen their collective effort and contribute to the establishment of a formal CoP. These activities included:

• Connect with those who were identified as central to the network, yet had never attended a meeting, and endeavour to address this groups needs directly.

• Identify where common-interests exist among researchers in each of the three identified teams (clusters) and connect them in an effort to build a more cohesive network.

• Follow up with those individuals identified as part of the network, but who have never attended a WATI meeting to gain a clear picture of the totality of the WATI research community.

Identification of individuals beyond this WATI group (i.e., the network's periphery) can be achieved in part using reputational sampling [57,58]. Similar to snowball sampling, reputational sampling is an iterative process that relies on the cumulative knowledge of network participants about who is involved in the network. This means engaging those identified by our participants who were not present at the WATI meeting and asking them the same questions. In doing so, a more complete picture of the network is produced.

## Conclusion

Community building, like any journey, is best done when there is a map to guide you and the willingness of people to travel in the same direction. This strategy piloted here provides a means to provide a map and assess the willingness, capabilities and KTE activities of people engaged in collaborative work. It often takes multiple attempts to form a functioning community of practice, despite the best efforts of community organizers [53]. Yet, we believe the process of building a map with WATI participants and exploring the motives for collaboration prior to any CoP activity itself increased receptiveness to fostering collective action among the WATI meeting attendees. The next step is to build on this early work to support the WATI initiative in moving beyond Wenger's *Coalescing *stage to the *Maturing *stage and to evaluate that progress as it unfolds. Such evaluation will determine the long-term impact that CoP-building has on KTE and eventually on the translation of WATI innovation into improved tobacco control practice.

## Abbreviations

CoP: Community of practice

KTE: Knowledge transfer and exchange

WATI: Web-assisted tobacco interventions

## Competing interests

The author(s) declare that they have no competing interests.

## Authors' contributions

Both authors equally contributed to the conceptualization of the study, its execution, and the overall analysis of the data. CN took the lead in developing the narrative for the manuscript and the preparation of tables. TH took the lead in the preparation of figures for the manuscript. CN coordinated the editing of the manuscript.
